# Changes in the Fluorescence Tracking of NaV1.6 Protein Expression in a BTBR T+Itpr3tf/J Autistic Mouse Model

**DOI:** 10.1155/2019/4893103

**Published:** 2019-12-17

**Authors:** Musaad A. Alshammari, Mohammad R. Khan, Fawaz Alasmari, Abdulaziz O. Alshehri, Rizwan Ali, Mohamed Boudjelal, Khalid A. Alhosaini, Abdurahman A. Niazy, Tahani K. Alshammari

**Affiliations:** ^1^Department of Pharmacology and Toxicology, College of Pharmacy, King Saud University, Saudi Arabia; ^2^Prince Naif Bin Abdulaziz Health Research Center (PNHRC), King Saud University, Saudi Arabia; ^3^Medical Research Core Facilities & Platforms, King Abdullah International Medical Research Center, Saudi Arabia; ^4^Department of Oral Medicine and Diagnostic Sciences, College of Dentistry, King Saud University, Saudi Arabia

## Abstract

The axon initial segment (AIS), the site of action potential initiation in neurons, is a critical determinant of neuronal excitability. Growing evidence indicates that appropriate recruitment of the AIS macrocomplex is essential for synchronized firing. However, disruption of the AIS structure is linked to the etiology of multiple disorders, including autism spectrum disorder (ASD), a condition characterized by deficits in social communication, stereotyped behaviors, and very limited interests. To date, a complete understanding of the molecular components that underlie the AIS in ASD has remained elusive. In this research, we examined the AIS structure in a BTBR T+Itpr3tf/J mouse model (BTBR), a valid model that exhibits behavioral, electrical, and molecular features of autism, and compared this to the C57BL/6J wild-type control mouse. Using Western blot studies and high-resolution confocal microscopy in the prefrontal frontal cortex (PFC), our data indicate disrupted expression of different isoforms of the voltage-gated sodium channels (NaV) at the AIS, whereas other components of AIS such as ankyrin-G and fibroblast growth factor 14 (FGF14) and contactin-associated protein 1 (Caspr) in BTBR were comparable to those in wild-type control mice. A Western blot assay showed that BTBR mice exhibited a marked increase in different sodium channel isoforms in the PFC compared to wild-type mice. Our results provide potential evidence for previously undescribed mechanisms that may play a role in the pathogenesis of autistic-like phenotypes in BTBR mice.

## 1. Introduction

Autism spectrum disorder (ASD) refers to a heterogeneous and indistinctly defined neurodevelopmental and neurobehavioral disorder involving deficits in social interaction, impairments in communication, and repetitive stereotyped patterns of behaviors and interests. However, the exact cause of ASD is not yet known. Genetic, epigenetic, or environmental factors are thought to underlie the pathogenesis of ASD and are currently being investigated [1]. The use of animal models of ASD will, therefore, provide important knowledge of behavioral phenotypes, underlying pathophysiology, molecular motives, and therapeutic developments [1, 2].

Phenotypic variations of this disorder have been identified in several mouse models parallel to the different mutations present in human ASD, including pharmacologically induced mice, valproic acid-induced mice, Shank3B mutant mice, and BTBR T+Itpr3tf/J (BTBR). Following the discovery of an association between prenatal exposure to valproic acid (VPA) and an elevated risk of ASD, the VPA-induced model has been utilized preclinically as an ASD model [3]. Conversely, SHANK3 mutations are highly prevalent in ASD patients, and the Shank3B mouse model has been extensively studied. In molecular terms, these mice exhibit deficits in neurotransmission, synaptic plasticity, and neuronal wiring. Behaviorally, they display core features of autistic-like behavior such as compulsive stereotyped repetitive behavior and reduced sociability [4]. In addition to the pharmacologically induced VPA model and the Shank3B genetic model, the BTBR inbred mouse strain is another valid model of ASD that has been used to represent idiopathic autism. The BTBR model displays various genetic, neuroanatomic, and molecular irregularities [5], including altered neurotrophic brain-derived factor (BDNF), the absence of the corpus callosum, and an imbalance in the excitatory/inhibitory (E/I) ratio [6]. Furthermore, the BTBR exhibits three unique and vigorous behavioral features that characterize ASD: deficits in social communication among both the young and adults, an uncommon ultrasonic utterance in newborns, and recurring fixed grooming behaviors [7–10]. Neuroimaging studies also indicate that altered neuronal activation and cognitive capacity evident in the BTBR mouse model may indicate a decreased cerebral blood flow and metabolism of cerebral oxygen [11]. These factors suggest that BTBR is a valid preclinical model that can be used to investigate the pathology of ASD.

Previously, it has been suggested that the developmental deregulation of neuronal networks due to postnatal events, including cell differentiation, synaptic formation, and plasticity, promotes autistic behavior in humans [12–15]. However, our understanding of the molecular neurobiological mechanisms that underlie ASD is far from complete [16].

The axon initial segment (AIS) is a very small subcellular structure that originates at a transient length from the neuronal soma immediately after the axon hillock [17]. It is enriched with scaffold proteins and voltage-gated sodium channels (NaV). It has been shown that different isoforms of NaV type 1 *α* subunits (NaV1) are concentrated in the AIS [18, 19]. This provides an increased flow rate of sodium ions (Na^+^) and a decreased action potential threshold [20]. NaV channels are distributed differentially at the AIS; for example, the NaV1.6 is localized in the distal part of the AIS, whereas the NaV1.2 is located at the proximal part of the AIS [21]. Altered structure and/or function of NaV1 *α* subunits in the AIS can also result in severe CNS disabilities. For instance, it has been found that missense alterations in the gene that encodes the NaV1.1 (SCN1A) may lead to serious severe epilepsy in infants due to imperfections at the level of the AIS [22]. Similarly, mutations in NaV1.2 (SCN2A) and NaV*β*1 (SCN1B) can lead to generalized epilepsies [23, 24]. Increased levels of expression of the NaV1.6 and ankyrin-G have also been reported at the AIS in an epileptic animal model [25]. Any alteration in intrinsic membrane proteins at the AIS might also aggravate the pathophysiological process of Angelman syndrome in a mouse model [26]. Notably, mutations and dysfunctions in genes encoding sodium channel isoforms are risk factors for ASD [27, 28].

Ankyrin-G protein has a significant physiological role in modulating the structure and function of ion channels in different types of cells [29]. The cytoskeletal scaffold protein ankyrin-G is a master organizer of membrane proteins in many types of cells [30]. Ankyrin-G (ANK3) has a pivotal role in ion channel targeting within neuronal populations [31]. In neurons, ankyrin-G is restricted to the AIS [32]. In vitro studies have shown that AIS proteins and ion channels not anchored by ankyrin-G are removed from the membrane by endocytosis [33]. Thus, the clustering of AIS membrane proteins depends on ankyrin-G [34]. Indeed, a lack of ankyrin-G expression in mice disrupts many accessory proteins clustering at the AIS, such as NaV channels [35, 36]. Therefore, all the AIS proteins are anchored directly or indirectly to the scaffold made of the cytoskeletal network, and as a result, ankyrin-G organizes the subcellular polarity of these protein molecules [37]. Genome-wide studies have also identified links between mutations in the gene encoding ankyrin-G, ANK3 gene, and ASD [38–41]. This implies that an alteration in AIS components is a molecular signature in the pathophysiology of ASD.

Abundant evidence has demonstrated that secreted fibroblast growth factors and their FGF tyrosine-kinase receptors (FGFRs) are essential in proper brain development, and for that reason, it is implicated in the pathology of various brain disorders. Secreted FGF2 is considered to be an indicator of allostatic load. It plays several roles in cell proliferation, differentiation, growth, survival, and angiogenesis [42, 43]. In a recent study, the level of FGF2 was reduced in the serum of ASD patients [44]. However, whether other nonsecreted members of the same gene family play a role in this process is not completely understood. In contrast to secreted FGFs, FGF14 belongs to the intracellular FGF group (iFGF) iFGF11 subfamily. This set of FGF members does not act through FGFRs [45, 46]. However, iFGFs control neuronal excitability and synaptic transmission by interacting intracellularly with the carboxy-terminal tail of NaV channels, axonal trafficking proteins (MAP kinase scaffolding protein, IB2 (MAPK8IP2)), and microtubules [47]. FGF14 is found in a range of neuronal tissues and present in gradient form in the AIS of the cultured hippocampal neurons [46]. It has been found to interact with NaV1.1, NaV1.2, and NaV1.6 channels directly via NaV C-termini [46, 48, 49]. Moreover, selected deletion of FGF14 has been implicated in deficits in neuronal excitability [50], immature dentate gyrus endophenotypes [51], and cognitive functions [52].

Although behavioral phenotypes of BTBR as an ASD animal model are well documented, at the molecular level, there are gaps in our knowledge [6, 7, 53–55], especially regarding the AIS structure. In this study, we examined different components of AIS in the prefrontal cortex brain region in the BTBR mouse model using high-resolution confocal imaging, image analysis, and Western blot studies. Our findings provide clear insights into the neuroaxonal structure and improve our understanding of the mechanisms of ASD.

## 2. Material and Methods

### 2.1. Animals

BTBR and C57BL/6J (wild-type) (obtained from Jackson Laboratory, Bar Harbor, ME, USA) male mice were maintained through an inbred background of backcrossing to littermate mating. Adult male mice at 2–4 months of age were used in this study. They were maintained at 25 ± 2°C in a 12 h light/dark cycle, housed in a hygienic environment, and fed with a Purina standard rodent chow diet (Grain Silos and Flour Mills Organization, Riyadh, Saudi Arabia). The mice were housed *n* ≤ 4-6 per cage and provided with water *ad libitum*. We utilized 4-6 per group. All experimental procedures were performed in accordance with the guidelines of the King Saud University Institutional Research Ethics Committee (REC).

### 2.2. Preparation of Brain Sections

Preparation and staining of mouse brain sections were previously described in [56]. In brief, the mice were deeply anesthetized with 10 : 1 mixture of ketamine 50 mg/ml (Tekam) and xylazine 20 mg/ml (Seton) (dose 0.1 ml/10 gm i.p.) diluted in 1X phosphate-buffered saline (PBS, pH = 7.4) (1X PBS) and then briefly perfused intracardially (flow rate: 8-10 ml/min for 2-5 min) with 1X PBS. This was followed by 10 min of 4% paraformaldehyde freshly prepared (Sigma-Aldrich) or commercially available 4% formaldehyde (a dilution of 37% formaldehyde solution, Sigma-Aldrich, in 1X PBS); all solutions were adjusted to pH 7.4. To ensure complete tissue fixation, brains were removed carefully and postfixed into the same fixative for 1 h at 4°C and then cryopreserved in 20-30% sucrose/PBS at 4°C in preparation for sectioning. Brains were then embedded in OCT compound (Tissue-Tek®, Ted Pella, Inc.) and sectioned sagittally into 14-20 *μ*m thick slices at -20°C using a Leica CM3050 S cryostat (Leica Microsystems). They were then mounted on glass slides (Fisher Scientific) and stored at -80°C for further use in cresyl and immunofluorescence studies. The second group of brains was gently dissected to isolate the frontal cortex according to the mouse brain atlas [57], and the samples were stored at -80°C for a Western blotting assay.

### 2.3. Immunofluorescence

Sagittal brain sections were washed with 1X PBS, incubated with a permeabilizing agent (1X Triton, 0.5X Tween in PBS, or -20°C acetone), then washed extensively with 1X PBS, and incubated with 5% normal goat serum NGS (Sigma-Aldrich) in 1X TBS containing 0.3% Triton X-100 for 1 h. This was followed by overnight incubation at 4°C with primary antibodies in 3% bovine serum albumin (BSA, Sigma-Aldrich) and 1X PBS containing 0.1% Tween 20. The primary antibodies used were mouse anti-FGF14 (1 : 300, NeuroMab, catalog number 75-096), mouse anti-ankyrin-G (1 : 1000, NeuroMab, catalog number 75-146), guinea pig anti-NeuN (1 : 750, Synaptic System, catalog number 266 004), mouse anti-NaV1.1 (1 : 500, NeuroMab, catalog number 75-023), mouse anti-NaV1.2 (1 : 300, NeuroMab, catalog number 75-024), mouse anti-NaV1.6 (1 : 300, NeuroMab, catalog number 75-026), mouse anti-Caspr (1 : 500, NeuroMab, catalog number 75-001), and mouse anti-PanNaV1 (1 : 300, NeuroMab, catalog number 75-405). After 12 hours of incubation, the sections were washed with 1X PBS and then incubated with appropriate secondary antibodies (1 : 250, Invitrogen) for 1 h in a 1X PBS solution containing 3% BSA and 0.1% Tween 20. Following incubation with secondary antibodies, the tissue was washed five more times with 1X PBS buffer solution. Finally, the glass slides were placed in an oven at 30-32°C for 10-15 min to dry and then coverslipped using Fisherfinest® Premium Cover Glass (Fisher Scientific) with ProLong® Gold antifade or ProLong® Gold antifade mountant with DAPI reagents (Life Technologies, catalog number P36941).

### 2.4. Confocal Microscopy

Confocal images were acquired using a Zeiss LSM-780 confocal microscope with a Plan Apochromat (63x/1.46 oil) objective. Multitrack acquisition was performed with excitation lines at 488 nm for Alexa 488, 543 nm for Alexa 568/594, and 633 nm for Alexa 633. *Z*-series stack confocal images were taken at fixed intervals with the same pinhole setting for all the three channels; the frame size was 1024 × 1024 pixels. Laser intensity and gain were kept constant for all the experimental groups.

### 2.5. Image Acquisition and Analysis

All confocal images were analyzed using ImageJ (NIH, USA, http://imagej.nih.gov/ij). For soma fluorescence intensity analysis, *Z*-stacks of confocal images were sum-projected, an ROI corresponding to soma was highlighted using an intensity threshold method, and mean fluorescence intensity was quantified. Quantification of the fluorescence intensities of AIS staining was performed using a method previously described in [47]. In brief, the fluorescence intensity of the AIS was analyzed by drawing a segmented line of 6 pixels in width along the AIS, starting from the soma and using anti-ankyrin-G or FGF14 staining as a marker of AIS location on the overlay image.

### 2.6. Western Blot

Western immunoblotting was performed as previously described in [58]. This involved homogenizing 50 mg of PFC from the brain tissues of BTBR and wild-type mice (*n* = 4-5 mice per group) in 0.32 M sucrose solution in the presence of protease and phosphatase inhibitors. For the Western blot, protein concentrations of samples from brain tissue homogenate were measured using a NanoDrop™ 8000 Spectrophotometer (Thermo Fisher Scientific), then prepared with 4X sodium dodecyl sulfate (SDS) and tris(2-carboxyethyl)phosphine (TCEP) (1 : 20), denatured for 10 min at 95°C, and then ran on 7.5% SDS-PAGE gel. The proteins were electrophoretically transferred from a gel onto a nitrocellulose membrane. Membranes were then blocked with 5% nonfat dry milk in TBS-T for 30 min and probed with the following primary antibodies in a blocking solution: anti-PanNaV1 (1 : 500, NeuroMab), anti-NaV1.1 (1 : 500, NeuroMab), anti-NaV1.2 (1 : 500, NeuroMab), anti-NaV1.6 (1 : 500, NeuroMab), mouse anti-ankyrin-G (1 : 500, NeuroMab), mouse anti-FGF14 (1 : 500, NeuroMab), and anti-Caspr (1 : 500, NeuroMab). This was followed by treatment with horseradish peroxidase-conjugated secondary antibodies and ECL Western blotting detection reagents. The signals were detected and measured with a luminescent image analyzer (ChemiDoc™ MP, Bio-Rad). ImageJ was used to measure the intensity of the proteins of interest.

### 2.7. Statistical Analysis

The data were presented as the mean ± standard error of the mean (SEM). The statistical significance of observed differences among groups was determined using two-sample Student's *t*-test or the corresponding nonparametric test, Mann–Whitney rank sum, based on the distribution of the samples underlying the populations. A level of *p* < 0.05 was considered statistically significant. Statistical analysis was performed using InStat, GraphPad Software, Inc., and SigmaPlot 12 (Systat Software, San Jose, CA). The data were tabulated in Microsoft Excel.

## 3. Results

### 3.1. Voltage-Gated Sodium Channel (NaV1.6, NaV1.2, and NaV1.1) Immunoreactivity and Expression in the BTBR Mouse PFC

**Figure 1:**
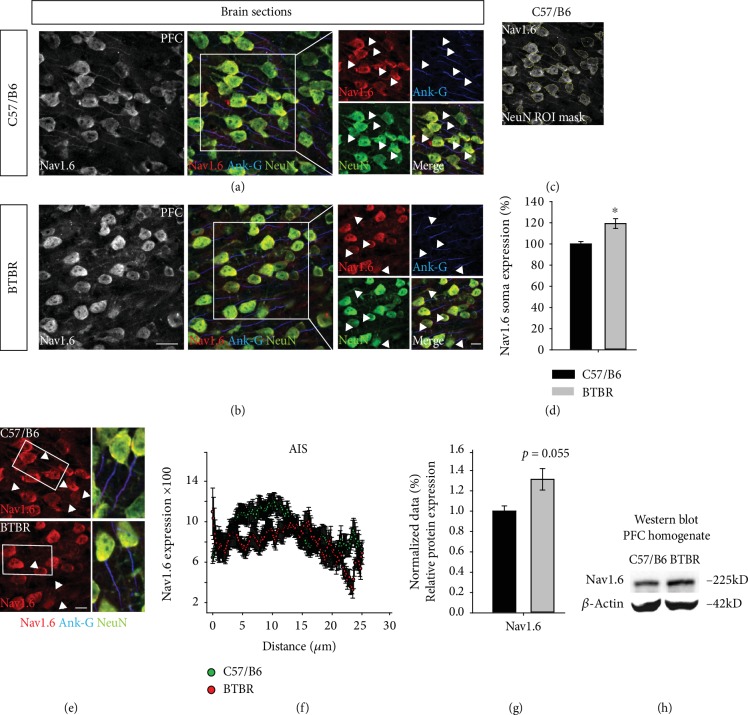
Altered NaV1.6 expression and distribution in the PFC of the BTBR mouse model. (a, b) The red channel represents confocal images of NaV1.6 (antisodium channel), the blue channel represents ankyrin-G (Ank-G), and the green channel represents NeuN immunofluorescence in the PFC of C57BL/6J and BTBR mice. (c) The mask ROI was used to detect NaV1.6 expression within the NeuN soma. (d) The quantification of NaV1.6 immunofluorescence expression within the soma of C57BL/6J and BTBR mice. *n* = 3 mice per group. (e) Representative confocal images of NaV1.6 (red) at the AIS in C57BL/6J and BTBR mice. (f) AIS tracking analysis of NaV1.6 in C57BL/6J and BTBR mice. *n* = 3 mice per group. (g) NaV1.6 protein levels in PFC lysates from C57BL/6J and BTBR mice and the Western blot bands. The protein expression was normalized with *β*-actin. *n* = 4-5 mice per group. Data represent mean ± SEM; statistical differences were assessed using Student's *t*-test (^∗^*p* < 0.05). Scale bars represent 20 *μ*m in (b) and 10 *μ*m in the white box (zoom images in (b)) and (e).

**Figure 2:**
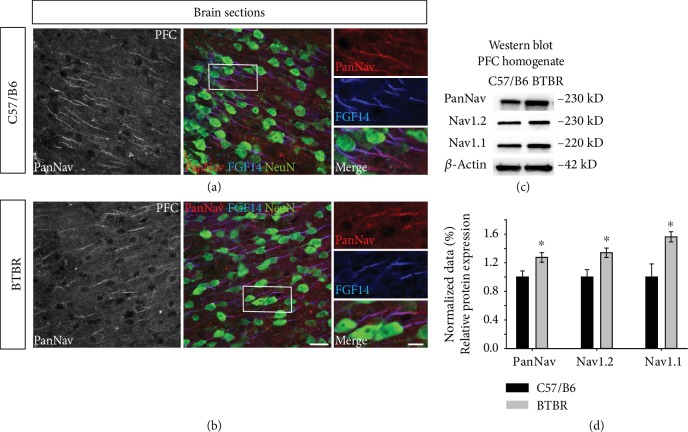
PanNaV, NaV1.1, and NaV1.2 expression in the PFC of the BTBR mouse model. (a, b) High-resolution confocal images where the red channel represents confocal images of PanNaV, the blue channel represents FGF14, and the green channel represents NeuN immunofluorescence in the PFC of C57BL/6J and BTBR mice. (c) Western blot analysis for PanNaV, NaV1.1, and NaV1.2 was performed on PFC homogenate and quantified. (d) The upregulation of PanNaV, NaV1.1, and NaV1.2. The protein expression was normalized with *β*-actin. Data represent mean ± SEM; statistical differences were assessed using Student's *t*-test (^∗^*p* < 0.05, *n* = 4-5 mice per group). Scale bars represent 20 *μ*m in (b) and 10 *μ*m in zoom images in (b).

We then examined NaV1.6 in the PFC—layer III—of BTBR, where immunofluorescence studies indicated that fluorescence expression in the soma of BTBR significantly increased compared to that of the wild-type mice (Figure 1(a)–(d), 119.25% ± 4.66 in BTBR vs. 100% ± 2.33 in wild type, *p* < 0.05, *n* = 3). Similarly, the Western blot analysis of the total protein homogenate of PFC indicated that NaV1.6 protein expression was elevated, albeit not significantly, in BTBR compared to the wild-type mice (Figure 1(g) and (h), 1.31 ± 0.105 in BTBR vs. 1.00 ± 0.051 in wild type, *p* = 0.056, *n* = 4-5). In the fluorescence tracking of axonal expression using ankyrin-G as an axonal marker, we found an aberrant pattern of distribution through the AIS structure (Figure 1(e) and (f)). Although it is difficult to anticipate the consequences of these molecular changes since the cortical circuitry is fairly complex [59], these findings indicate that NaV1.6 functionality could be altered in BTBR mice. This led us to investigate other isoforms of NaV1 *α* subunits. Thus, we examined NaV1.1 and NaV1.2 using the Western blot and found that, in the PFC of BTBR, both isoforms were elevated significantly compared to the wild type controls. In support of these findings, the expression of PanNaV increased in the PFC of BTBR compared to wild-type mice (Figure 2(c) and (d), 1.27 ± 0.067 in BTBR vs. 1.00 ± 0.085 in wild type, *p* < 0.05, for PanNaV; 1.33 ± 0.066 in BTBR vs. 1.00 ± 0.103 in wild type, *p* < 0.05, for NaV1.2; and 1.56 ± 0.071 in BTBR vs. 1.00 ± 0.183 in wild type, *p* < 0.05, for NaV1.1; *n* = 4-5 per group). These results showed a disruption in NaV1.6, NaV1.2, and NaV1.1 expressions, which could be implicated in altered firing and the synaptic wiring in ASD.

### 3.2. Ankyrin-G Expression in the BTBR Mouse PFC

**Figure 3:**
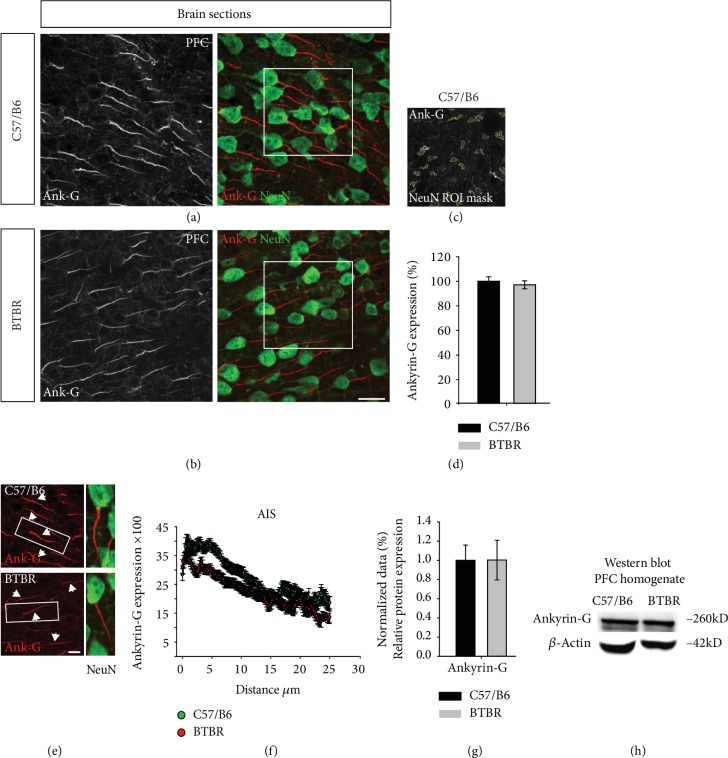
Ankyrin-G expression in the PFC of the BTBR mouse model. (a, b) High-resolution confocal images acquired from the PFC of C57BL/6J and BTBR: ankyrin-G (grey and red) and NeuN (green). (c) The ROI mask used to quantify ankyrin-G. (d) The quantification of ankyrin-G immunofluorescence. *n* = 3 mice per group. (e) Representative high-resolution confocal images of AIS in C57BL/6J and BTBR mice. (f) The AIS tracking analysis of ankyrin-G in C57BL/6J and BTBR mice. *n* = 3 mice per group. (g) Ankyrin-G protein levels in PFC lysates from C57BL/6J and BTBR mice and the Western blot bands. The protein expression was normalized with *β*-actin. *n* = 4-5 mice per group. Data represent mean ± SEM; statistical differences were assessed using Student's *t*-test (^∗^*p* < 0.05). Scale bars represent 20 *μ*m in (b) and 10 *μ*m in (e).

To explore the expression of the AIS scaffold protein ankyrin-G, we examined ankyrin-G in the soma of matured neurons in the PFC of BTBR and found that immunostaining appeared to have not been altered (Figure 3(a)–(d), 97.31% ± 3.203 in BTBR vs. 100% ± 3.573 in wild type, *p* = 0.512, *n* = 3 per group). To validate this finding, we examined the PFC total protein homogenate expression, consistent with immunofluorescence studies from layer III, and found that ankyrin-G was unchanged in the BTBR mouse model compared to the wild-type mice (Figure 3(g) and (h), 1.00% ± 0.206 in BTBR vs. 1.00 ± 0.158 in wild type, *p* = 0.991, *n* = 4-5 per group). The tracking of the AIS structure indicated that the ankyrin-G expression pattern is upregulated in the proximal part of the AIS (Figure 3(e) and (f)). These findings suggest that the cytoskeleton structure in BTBR mice might be unaffected.

### 3.3. Fibroblast Growth Factor 14 (FGF14) Expression in BTBR

**Figure 4:**
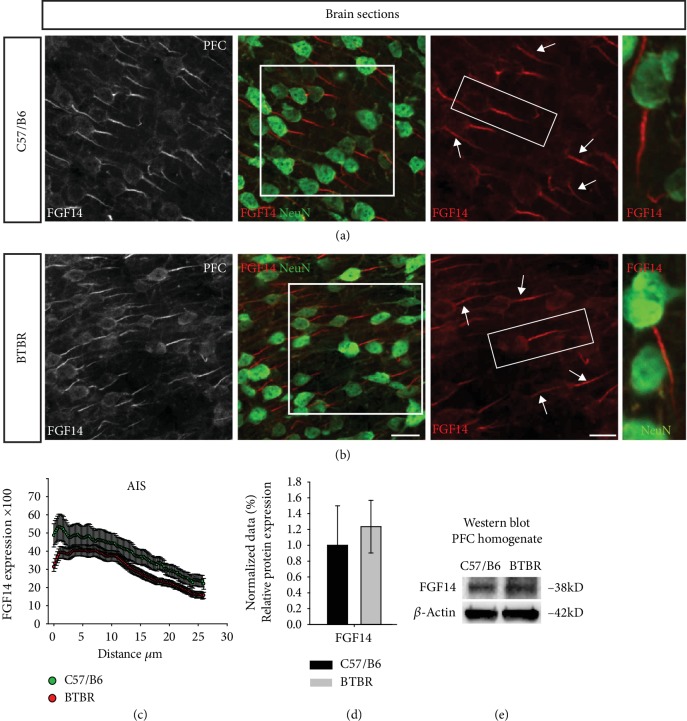
FGF14 expression in the AIS of the BTBR mouse PFC. (a, b) Representative confocal images of FGF14 (grey and red) and NeuN (green) from the PFC of C57BL/6J and BTBR; FGF14 (grey and red) and NeuN (green) arrows represent AIS. (c) The expression of FGF14 across the AIS. *n* = 3 mice per group. (d, e) Immunoblot detection of FGF14 in the PFC homogenate from C57BL/6J and BTBR and the quantitative Western blot analysis. *n* = 4-5 mice per group. Data represent mean ± SEM; statistical differences were assessed using Student's *t*-test (^∗^*p* < 0.05). Scale bars represent 20 *μ*m in (b).

Because most NaV1 *α* isoforms were disrupted, we continued with our studies and examined FGF14, a key regulator of NaV channels. The immunohistochemical staining examinations indicated that FGF14 expression was comparable in both types of mice (Figure 4(a) and (b)). These results were confirmed by Western blot analysis (Figure 4(d) and (e), 1.23 ± 0.332 in BTBR vs. 1.00 ± 0.499 in wild type, *p* = 0.705, *n* = 4-5 per group). The tracking of the AIS structure indicated an increase in FGF14 expression in the distal part of PFC neurons (Figure 4(c)). These results suggest that the increase in NaV1.6 expression in the AIS is accompanied by an increase in FGF14 in AIS.

### 3.4. Examination of the Node of Ranvier in the BTBR Mouse Model

**Figure 5:**
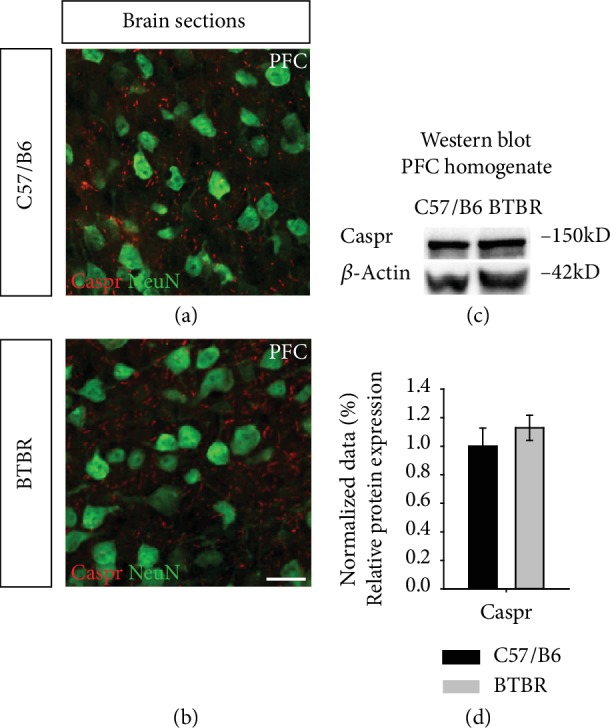
Caspr expression in the PFC of the BTBR mouse model. (a, b) Representative high-resolution confocal images of the PFC of BTBR and C57BL/6J control mice immunostained for Caspr (red) in combination with NeuN (green). (c) Immunoblot detection of Caspr in the PFC homogenate from C57BL/6J and BTBR mice. (d) The quantitative Western blot analysis normalized with *β*-actin. *n* = 4-5 mice per group. Data represent mean ± SEM; statistical differences were assessed using Student's *t*-test. Scale bars represent 20 *μ*m in (b).

We then examined a vital component of the node of Ranvier by analyzing the expression of Caspr in the PFC of BTBR mice. The Caspr immunoreactivity analysis of the PFC homogenate revealed nonsignificant Caspr expression in BTBR compared to wild-type mice (Figure 5(c) and (d), 1.12 ± 0.087 in BTBR vs. 1.00 ± 0.127 in wild type, *p* = 0.453, *n* = 4-5 per group). These results suggest that the node of Ranvier may be unaffected in the BTBR mouse model.

## 4. Discussion

Although studies have shown that ASD is characterized by a reduced excitation and inhibition ratio, examination of the AIS molecules may reveal the possible mechanism and etiology of ASD and therapeutic targets for its improvement [60]. The AIS-specific proteins play an essential role in the physiology and the function of neuronal populations [61].

In the current study, our aim was to ascertain whether the disruption of AIS accessory proteins is observed in BTBR mice, as this feature could be a molecular signature of ASD pathogenesis. Our results showed that the expression of different NaV1 *α* isoforms was reduced and that the AIS accessory scaffold protein ankyrin-G was not altered. Additionally, FGF14 expression was comparable in BTBR and wild-type controls. Besides, the expression level of Caspr, a key molecule of the node of Ranvier, was not altered in BTBR compared to the wild-type controls, indicating that neuronal firing could be affected in this model.

The BTBR was derived from the inbred strain Black and Tan Brachyury (BTBR), which carries mutations in a couple of genes including Itpr3 (inositol 1,4,5-trisphosphate receptor 3) and T (brachyury) genes [62]. This strain has exhibited most of the behavioral disabilities observed in ASD. Therefore, these mice could result as a valid model in investigating the role played by the structural proteins of AIS in the cortical tissue of the autistic brain.

In our experimental setup, we conducted the immunofluorescence studies in layer III of the prefrontal cortex, while the Western blotting analysis was performed on the whole homogenate of the prefrontal cortex. This route was taken because, based on our experimental experience, most AIS were condensed and easily detected in layer III of the prefrontal cortex. Additionally, it was reported that within the cortex, the deep III layer was characterized by the existence of condensed axonal projections [63].

There is a high degree of enrichment and subcellular polarity for NaV channels at the AIS, which facilitates high Na^+^ current density and a low action potential threshold [20]. NaV1.6 is the major NaV1 *α* subunit, which is expressed either with NaV1.1/NaV1.2 or alone in different subdomains of AIS in different neurons [21, 64, 65].

Nav1.6 is a prime voltage-gated sodium channel in the brain [66]. The AIS is enriched by this isoform, which enables it to modulate the initiation of action potentials [67]. It is also concentrated at the nodes of Ranvier and other subcellular structures, including the soma and the dendrites [66].

NaV1.6 plays a critical role in the generation of persistent and resurgent cellular currents. Thus, altered expression in this protein could have serious consequences [66]. Mutations in genes encoding different isoforms of sodium channels will result in ASD in humans, and different single-nucleotide polymorphisms (SNPs) have been found to be associated with ASD [68]. In this research, we found that the expression of NaV1.6 increased in the soma and the AIS, suggesting that both persistent and resurgent currents could be altered. It is quite difficult to relate alterations in some sodium channel isoforms to system-level dysfunction of neural wiring. It is for future studies to characterize the axonal structure of inhibitory interneurons and analyze the functional characteristics of neuronal populations using electrophysiological recordings.

The accumulation of NaV1.2 at the proximal AIS promotes action potential propagation to the soma and sets the action potential threshold of the somatodendritic region of the neuron. The NaV1.2 channel may control action potential backpropagation because of its high density at the proximal AIS. Our findings demonstrated an alteration in the expression of NaV1.2 channels in the AIS of the cortical neurons of BTBR mice, suggesting that this may contribute to the functional alterations observed in ASD [21]. Mutations in NaV1.2 may cause common epilepsies [23]. Similarly, a recent genomic study indicates that mutations in the NaV1.2 gene (SCN2A) in ASD are a consistent finding and may be considered an ASD risk factor [27]. Small changes in the density of NaV channels could change the excitability of a neuron, and reduced integrity of the AIS barrier would be expected to affect the normal distribution of axonal and somatodendritic proteins in the cell [34]. The increased expression of NaV1.2 observed in our studies may not necessarily indicate that more excitation is taking place. It may be a compensatory mechanism as the increased production of protein could be attributed to either improper positions, lack of function, or even a deficit in maintenance. Importantly, alterations of the functioning of NaV channels at the AIS may cause severe CNS dysfunction. A recent study indicated that variants in the NaV1.2-encoding gene lead to a gain in function and an increase in neuronal excitability: these result in an imbalance in the excitation/inhibition (E/I) ratio and seizures [27].

In addition to altered NaV1.2 and NaV1.6, our analysis indicated an increase in NaV1.1, suggesting that the inhibitory current is also altered [69]. Increased NaV1.1 was previously reported in the dorsal root ganglion following nerve injury, indicating that it could be a mechanism in modulating neuropathic pain [70]. The NaV1.1 (SCN1A) locus was identified as indicating susceptibility to autism during genome-wide association studies [71, 72]. The mutations in the NaV1.1 gene were identified in individuals with familial autism by genome sequencing [68]. The missense mutations in the gene encoding NaV1.1 can lead to severe epilepsy in infants due to defects at the level of the AIS [22]. It has also been reported that heterozygous mice with a missense mutation in the NaV1.1 channel develop hyperactivity, autistic traits, cognitive deficits, anxiety, social interaction deficits, and excessive stereotyped behaviors. These cognitive and behavioral deficits are caused by reduced action potential firing in GABAergic interneurons [73].

The molecular mechanisms that modulate the composite configuration of the NaV1 *α* subunit in the AIS are complex. An altered excitation over inhibition ratio is a unified hypothesis underlying ASD and related disorders. Strong evidence suggests that an increase in the ratio between excitation and inhibition, leading to the hyperexcitability of cortical circuits, is implicated in ASD. This circuitry imbalance leads to a deficit in learning and cognitive capacity as well as sociability [60].

We then moved on to examine ankyrin-G. The cytoskeletal adaptor protein ankyrin-G has a binding site for NaV1 *α* subunits. Therefore, we investigated whether BTBR mice would also exhibit altered Ank-G expression. We found that ankyrin-G immunostaining in the mature neurons of BTBR mice was comparable to that of wild-type mice. It has previously been reported that the cytoskeletal scaffold protein ankyrin-G is a principal architect of membrane proteins and subcellular polarity in different types of cells [32, 74–76]. In neurons, ankyrin-G is concentrated on the AIS and NaV channels that bind to ankyrin-G [32, 33]. The silencing of ankyrin-G gene expression by RNA interference in mice interferes with the assembly of NaV channels at the AIS [35, 36]. This suggests that mutations harbored in BTBR mice do not affect the initial steps in AIS assembly; however, they do affect the localization and recruitment of subsequent proteins, including sodium channels, at the AIS [31].

In this research, we found that FGF14 is prominently expressed in the AIS of mature cortical neurons. FGF14 and NaV immunostaining was disrupted in the AIS of cortical neurons in BTBR mice, indicating a regulatory interaction among these proteins. Similarly, a recent study showed that FGF14 plays a regulatory role in the localization of NaV1 *α* subunits in the Purkinje neuron in the AIS, and the expression of NaV1.6 was found to decrease over the length of the AIS of the Purkinje neuron [77].

The node of Ranvier has clear links to demyelinating diseases [78, 79]. It also has a common molecular organization to the AIS, probably because the node of Ranvier evolved from the AIS [34]. The genes that are translated into Caspr, which are enriched at the node of Ranvier, have been classified as a prime locus of susceptibility for autism spectrum disorders, bipolar disorder, and mental retardation [80, 81].

The mutation in the inositol triphosphate receptor is a key component in dysfunctional phenotypes observed in BTBR. Inositol triphosphate modulates various physiological processes by promoting cellular calcium signals [82]. A mutation in the inositol triphosphate receptor in BTBR might be responsible for the deficit in the trafficking of AIS components, leading to the molecular phenotypes observed in this study. In line with this evidence, a previous study has implicated Wnt/Ryk calcium signaling in the regulation of axonal outgrowth, axonal development, and guidance [83].

## 5. Conclusion and Future Directions

These results provide novel insights into the etiology of ASD and favor the notion that alterations in architectural proteins in the AIS of growing neurons are significant in the development of the autistic brain in BTBR mice. Future studies might be needed to address whether other components of AIS such as FGF-13, FGF-12, neurofascin, and *β*IV spectrin are altered in BTBR mice. It would also be useful to shed light on the functionality of voltage-gated sodium channel *α* subunits in the cortical region of the ASD model and characterize their electrophysiological features. This would help determine the functional architecture of AIS and establish neuronal polarity.

## Data Availability

The datasets used and/or analyzed during the current study are available from the corresponding author on reasonable request.
